# Durable nanocomposite face masks with high particulate filtration and rapid inactivation of coronaviruses

**DOI:** 10.1038/s41598-021-03771-1

**Published:** 2021-12-21

**Authors:** Andrew Gonzalez, Hamada A. Aboubakr, John Brockgreitens, Weixing Hao, Yang Wang, Sagar M. Goyal, Abdennour Abbas

**Affiliations:** 1 Claros Technologies Inc., 1000 Westgate Drive, Suite 1005, St. Paul, MN 55114 USA; 2 Department of Veterinary Population Medicine, University of Minnesota Twin Cities, 1333 Gortner Ave., St. Paul, MN 55108 USA; 3 Department of Civil, Architectural, and Environmental Engineering, Missouri University of Science and Technology, 1401 N Pine St., Rolla, MO 65409 USA; 4 Department of Bioproducts and Biosystems Engineering, University of Minnesota-Twin Cities, 2004 Folwell Ave, St. Paul, MN 55108 USA

**Keywords:** Nanoscale materials, Nanoparticles, Chemistry, Materials chemistry, Environmental, health and safety issues

## Abstract

The COVID-19 pandemic presents a unique challenge to the healthcare community due to the high infectivity rate and need for effective personal protective equipment. Zinc oxide nanoparticles have shown promising antimicrobial properties and are recognized as a safe additive in many food and cosmetic products. This work presents a novel nanocomposite synthesis approach, which allows zinc oxide nanoparticles to be grown within textile and face mask materials, including melt-blown polypropylene and nylon-cotton. The resulting nanocomposite achieves greater than 3 log_10_ reduction (≥ 99.9%) in coronavirus titer within a contact time of 10 min, by disintegrating the viral envelope. The new nanocomposite textile retains activity even after 100 laundry cycles and has been dermatologist tested as non-irritant and hypoallergenic. Various face mask designs were tested to improve filtration efficiency and breathability while offering antiviral protection, with Claros’ design reporting higher filtration efficiency than surgical masks (> 50%) for particles ranged 200 nm to 5 µm in size.

## Introduction

Nanotechnology is the study and utilization of materials on the nanometer scale (10^–9^ m). Because of the small size, these materials exhibit unique physical and chemical properties compared to their bulk counterparts and can be adapted for use in medicine, environmental remediation, and aerospace^1^. Metallic nanoparticles such as copper, tungsten, silver and their respective oxides are currently utilized for antimicrobial applications in consumer apparel to help fight bacteria that cause malodor^2^. The large surface area to volume ratio of these nanoparticles allows for an increased exposure of the active metal or metal oxide surface to bacteria, where metal ions can then penetrate and kill the bacteria^3^. Since the outbreak of the coronavirus disease-2019 (COVID-19) pandemic caused by the Severe Acute Respiratory Syndrome Coronavirus-2 (SARS-CoV-2), nanotechnology has been investigated for potential healthcare applications to provide protection, improve the diagnostics, and as a direct therapeutic treatment^4,5^. Research has been conducted to determine the antiviral properties of the metallic nanoparticles described above, with results showing good virucidal activity against specific viral strains, but potential cytotoxicity hazards depending on the concentration of the nanoparticle were reported^6–9^. Furthermore, common nanoparticles such as silver and copper have been shown to be harmful to the environment and aquatic wildlife due to particle leaching^10,11^. To combat this leaching effect, methods are needed that can bind the nanoparticles to the surface of the underlying substrate. The current state of the field relies on the deposition or coating of the substrate with pre-formed nanoparticles that are adhered to the surface through processes such as dip-coating^12–14^. Unfortunately, this adhesion is generally weak, and nanoparticles are released from the treated sample after repeated use or exposure to the environment^15–17^.

Zinc oxide is an attractive alternative to these pollutant materials due to its benign nature and ease of manufacturing. While less potent than silver or copper, zinc oxide nanoparticles still retain antibacterial properties because the Zn^2+^ ions released from the surface are able to penetrate the cell and alter its metabolic processes. In addition, sufficient concentrations of Zn^2+^ ions can kill the cells by generation of reactive oxygen species^18,19^. The antiviral mechanism is a similar process, whereby the Zn^2+^ ions can bind to the viral envelope or related proteins, impairing the binding of the virus to the host cell, resulting in the inactivation of the virus^9,20^. Zinc oxide is generally recognized as safe (GRAS) by the Food and Drug Administration (FDA), meaning it has been tested for safe use in consumer goods and has minimal impact on the environment^21^. Despite the safe nature of zinc oxide and zinc nanoparticles, high volumes that are shed from conventional coating processes could still have negative effects on human health and the environment, especially when inhaled through a face mask^22^. Utilizing an alternative synthesis method that embeds the nanoparticles inside of the mask material would improve performance and help protect the user and minimize shedding over time.

This work demonstrates a novel approach for nanoparticle formation, which results in a functional nanocomposite- a multi-phased material in which one of the constituent materials is in the nanoscale, typically a metal or non-metal nanoparticle phase in a macroscopic support material—that has improved durability and longevity over traditional surface coated materials. This technology can be applied to nearly any natural or synthetic fiber, making it ideal for functional textile applications. The process, known as “Crescoating^®^” (coating by growth [-*cresco*]) relies on an in-situ growth process from thermal treatment of a dissolved ionic precursor solution. The solution is impregnated into a support material followed by heating so as to begin nucleation and growth of nanoparticles within the support. Previous work has shown the efficacy of this method on polyurethane foam for environmental remediation, as well as natural and synthetic fibers for functional textiles^23–25^. In these materials, growth occurs within and on the surface of the substrate, and the nanoparticles become embedded in it, resulting in a nanocomposite material with improved durability and stability over traditional surface-treated products, which will leach nanoparticles over time and lose efficacy.

Due to the COVID-19 pandemic, surgical and cloth face masks, and other personal protective equipment (PPE) are being adopted as preventative measures to help limit the risk of infection and transmission. SARS-CoV-2 has become a hospital-acquired infection (HAI) and a major contributor to the proliferation of COVD-19, with over 570,000 infections and 2500 deaths among healthcare workers in the Americas alone^26^. This is largely explained by the fact that SARS-CoV-2 remains infectious on surfaces for 3–14 days as we and other teams have recently reported^27,28^. This results in the spread of infection by contact with personal protective equipment during wearing or disposal. The Crescoating^®^ synthesis process presents a new path for the fabrication of antiviral face masks that are inexpensive, durable, and effective at neutralizing the SARS-CoV-2 virus, thus upgrading PPE from simple physical barriers to active catalytic materials. Broadly, this process can be applied to any textile, such as lab coats, scrubs, bed sheets, and other common healthcare fabrics that would help to reduce HAIs.

## Results and discussions

### In-situ growth of nanoparticles for nanocomposite formation

**Figure 1:**
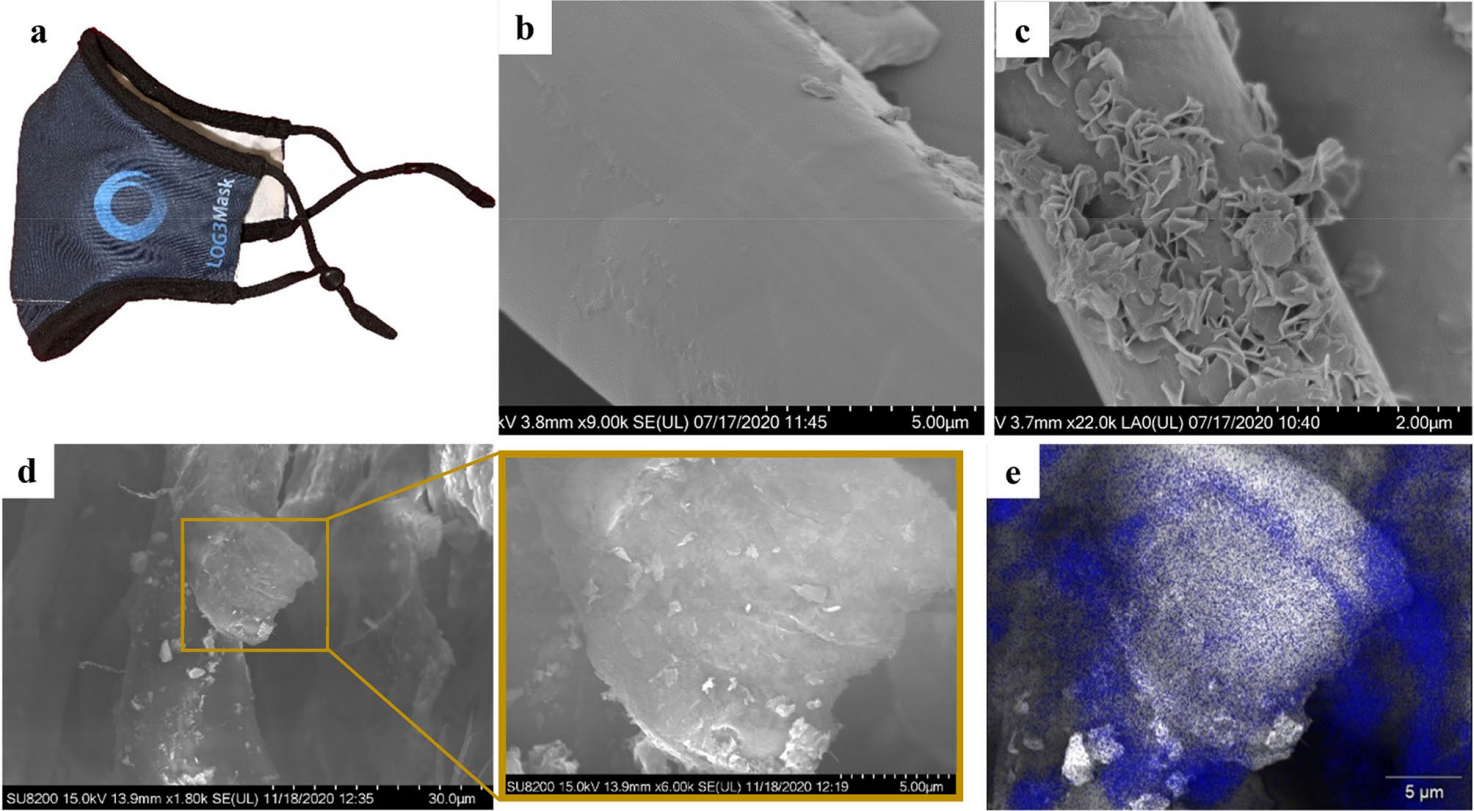
SEM images of a polypropylene facemask and polyester-cotton blend fabric before and after functionalization in a commercial oven. (**a**) Example of facemask designed by Claros Technologies, (**b**) untreated polypropylene textile, (**c**) zinc-polypropylene nanocomposite textile with “petal” shaped zinc particles, (**d**) polyester-cotton fabric at various magnifications showing internal nanoparticle growth. In image (**d**) the yellow boxes correspond to a fiber region which is magnified in the following image to show nanoparticle growth in the fiber cross-section. (**e**) Energy-dispersive X-ray spectroscopy (EDX) imaging of cross section image, (**d**) with zinc highlighted in blue.

In this study, fabrication of the virucidal facemasks named LOG3Mask, was performed by soaking the substrate, either polypropylene surgical masks or commercial cotton facemasks in a zinc salt solution. After immersion, the soaked sample is heated in a commercial oven at 100 °C to evaporate the water and initiate nucleation and growth of zinc oxide nanoparticles. The resulting nanoparticles or nanostructures are randomly distributed within and on the surface of the material and vary in shape and size from 5 to 500 nm, depending on the growth conditions. Figure 1 shows the growth of nanoparticles not only on the surface but also within the bulk of the fiber material.

Energy-dispersive X-ray spectroscopy (EDX) analysis shows that the nanoparticle loading within the fibers is usually half the loading on the surface, which is expected as nanoparticle growth inside the fibers is diffusion limited (Supplemental Fig. 3 and Table A).

**Figure 2:**
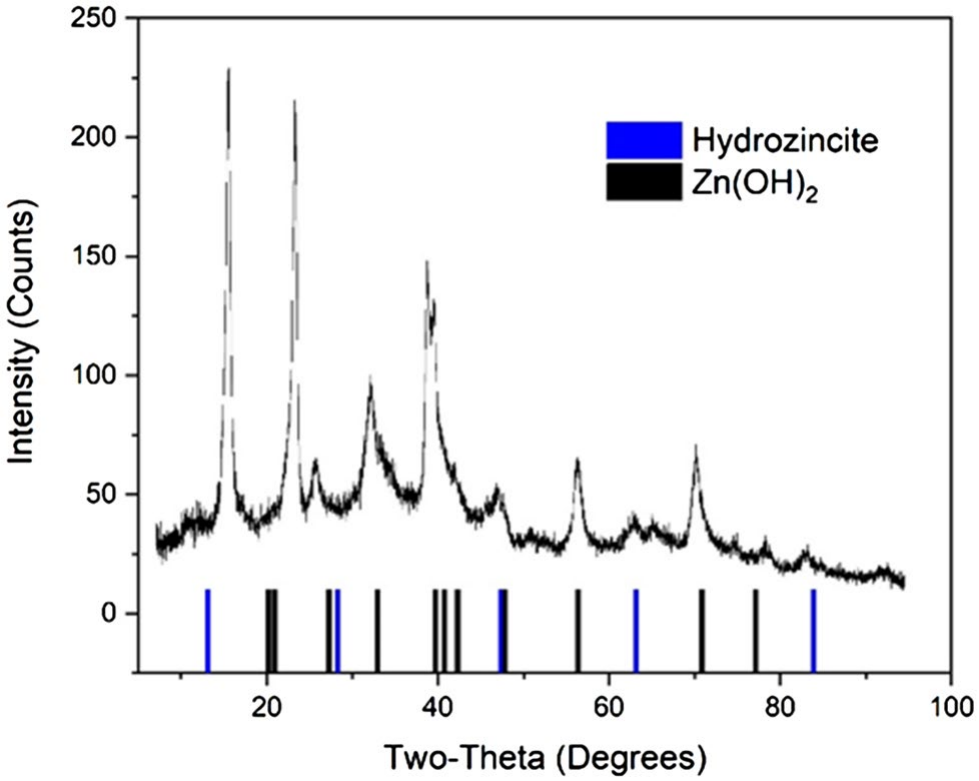
XRD analysis of the nanoparticles formed after heating the textiles at 100 °C shows a mixture of hydrozincite and zinc hydroxide present. These intermediates are the necessary precursors to zinc oxide formation through thermal decomposition.

The exact mechanism of nanoparticle formation involves the formation of a zinc hydroxide intermediate through hydrolysis of the zinc ions, which after significant heating decomposes into zinc oxide (see Supplemental Fig. 1), as we have previously reported^29^. The presence of zinc carbonate hydroxide and zinc hydroxide has also been detected on the samples through X-ray Diffraction (XRD) as shown in Fig. 2 and is likely a result of decomposition of the salt used.

All these products are categorized as Generally Recognized As Safe by the US-FDA and are used in commercial or healthcare applications^30^. Mass measurements before and after growth revealed a nanoparticle loading of 2–4% by mass of the final composite. Zinc colorimetry further revealed ionic zinc loading concentrations of 0.5–2.0% by mass, indicating that the zinc exists as a complexed material such as the oxide or hydroxide.

### Virucidal testing of resulting nanocomposite

**Figure 3:**
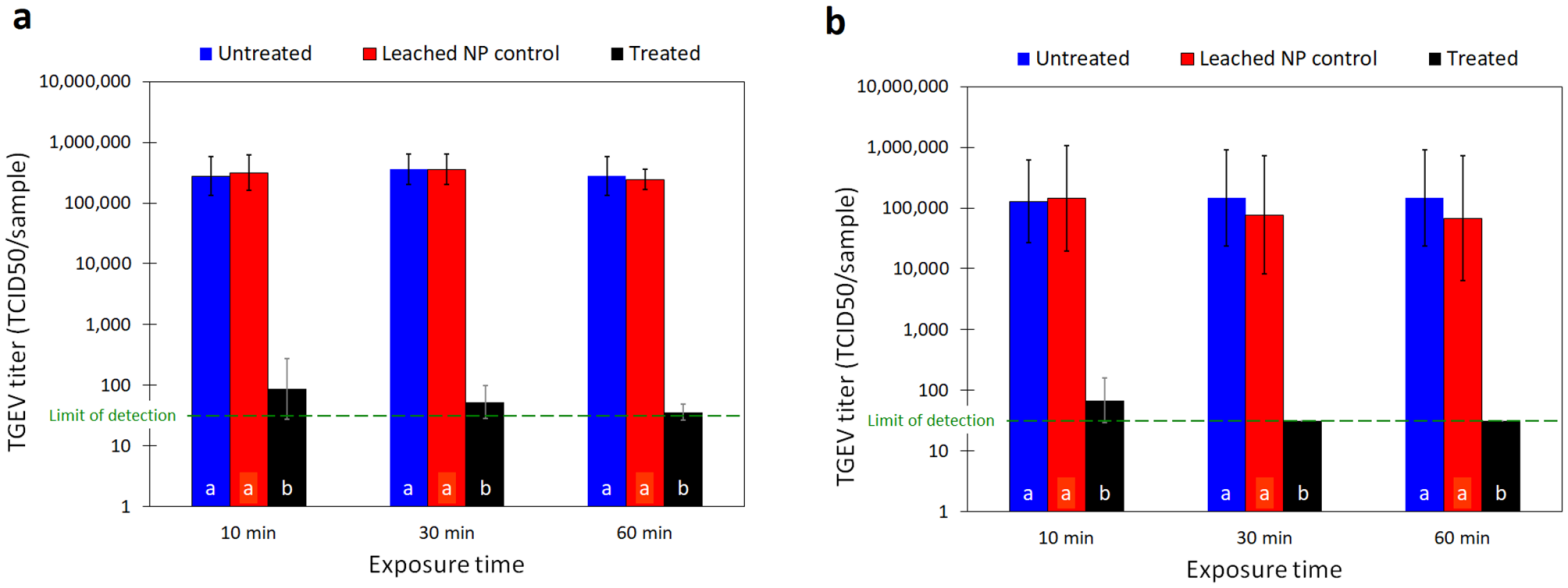
Titer of infectious TGEV particles recovered from (**a**) nylon-cotton fabric specimen and (**b**) face mask specimens after 10, 30, and 60 min contact times. The columns are the geometric mean of 6 replicates. The error bars represent ± one geometric standard deviation. The scattered green line is the limit of detection. Same letters at each column base indicate geometric means that are not significantly different from one another at each contact time *p* ≥ 0.05.

Virucidal properties of both untreated and nanocomposite materials (face mask and nylon/cotton fabrics) were measured using a slighly modified ISO 18184 method for determination of antiviral activity of textile products^31^. For this test we selected the transmissible gastroenteritis virus (TGEV, a porcine alpha coronavirus), as a surrogate of SARS-CoV-2. Breifly, we exposed TGEV to the nanocomposite materials (face mask and nylon/cotton fabrics) then recovered and titrated the surviving viral particles from the fabrics and titrated them using the 50% tissue culture infectious dose (TCID_50_) method (see “Methods” section for experimental details). Results of these experiments showed reduction in the titer of infectious virus of more than three orders of magnitude (≥ 99.9%) after only 10 min of exposure. Extended exposure times (30 and 60 min) showed statistically nonsignificant (*p* ≥ 0.05) increases in virus reductions for that puts the infectious virus titer below the limit of detection (> 4 log or 99.99% reduction) (Fig. 3a,b). This largely surpasses commercially available products that reported between 2 and 3 log reduction after 2 h of contact^31^. The virus suspension was prepared in Eagle’s minimal essential medium (MEM) containing 4% fetal bovine serum, which was considered as a high organic protein load. The strong virucidal efficacy of the nanocomposite materials despite the presence of high protein organic load indicates that this efficacy will not be affected by the protein-content of human’s sputum droplets in which SARS-CoV-2 is shed. When the virus nanocomposite materials were initially vortexed in the recovery solution before adding the virus to the recovery solution for 10, 30, and 60 min (leached nanoparticle control experiment), no virucidal efficacy was observed (Fig. 3a,b). These results confirm that the inactivation of the virus was solely due to the direct contact of the virus with the nanocomposite materials, and not as a result of any nanoparticles that might partially leach into the recovery solution. In addition, these results indicate the improved durability of the nanoparticle growth process over a traditional surface treatment, which can rapidly release particles into the environment.

**Figure 4:**
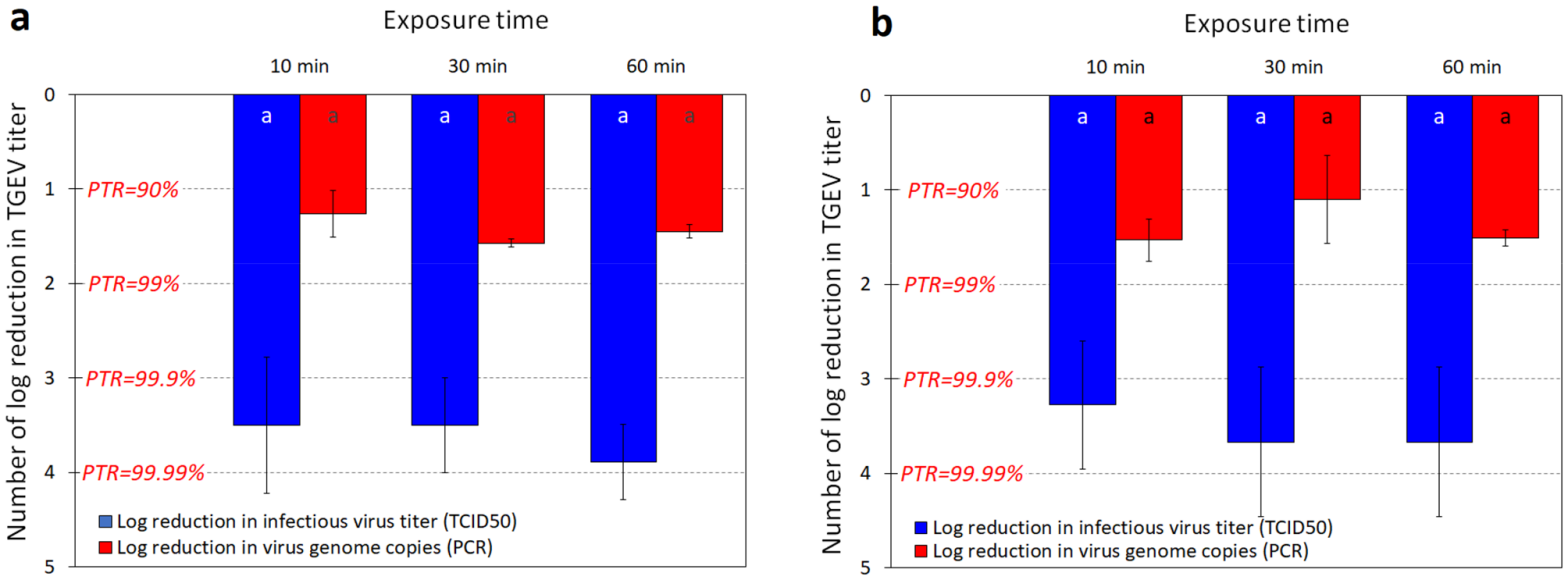
Number of log reduction in the infectious titer and viral genome copies after 10, 30, and 60 min contact times with (**a**) nylon-cotton fabric specimen and (**b**) face mask specimens. The columns are the arithmetic mean and the error bars represent ± one standard deviation. Same letters at each column base indicate geometric means that are not significantly different from one another at each contact time p ≥ 0.05. *PTR* percentage of virus titer reduction.

In another experiment, we used real-time reverse transcription quantitative polymerase chain reaction technique (RT-qPCR) to quantify the reduction in the viral genome copy numbers recovered from the spiked control and nanocomposite materials after 10, 30, and 60 min of contact. Lower reduction (approximately one order of magnitude) in viral genome copies was observed (Fig. 4a, b) as compared to the > 3 log reduction in the titer of infectious TGEV particle as measured by cell culture. This indicates that the majority of the neutralized viral particles were inactivated by the impact of nanoparticles on the viral envelope and its linked proteins. The fraction of viral genome that was reduced indicate that disintegration in the viral capsids occurred in approximately 1 log of the > 3 log inactivated viral particles during the first 10 min of contact with the treated fabrics.

As presented in Table 1, the virucidal activity of our nanocomposite face mask materials is either higher or faster than that of other suggested nanofunctionalized fabric face masks reported in litirature. None of the available materials can acheive > 4 log (99.99%) reduction in the virus load within 10 min contact time as ours does. The photoactive conjugated polymers in masks structure is the only available one that inactivates > 4 log reduction^32^. However, it requires at least 1 h contact time compared to 10 min and exposure to light near to UV wavelength as a virucidal-inducing treatment which gives additional advantage to our proposed materials that do not require any virucidal-inducing treatmets.

**Table 1:** Virucidal efficacy of other nanofunctional fabric face mask materials proposed in literature.

Technology	Active effect	Exposure time (h)	Virus	Log reduction	% Reduction	References
Graphene nanoplatelet and graphene oxide functionalization of face mask	Virus entraping in the mask	2	SARS-CoV- 2	2	99	De Maio et al.^33^
Silver nanoparticles	Silver nanoparticles	1	SARS-CoV-2	NR	21.40	Hamouda et al.^34^
Fabric mask composed of polyamide66 layer embedded with Zn(II)	Capturing the virus by PA66 and Killing the virus by Zn(II)	1	SARS-CoV-2	2	99	Gopal et al.^35^
Dip coating of PP in Cu@ZIF-8 NWs dispersion	Biocidal effect of Cu(II) and Zn(II)	48	SARS-CoV-2	< 1	55	Kumar et al.^36^
Photoactive conjugated polymers in masks structure	Release biocidal ROS when exposed to visible light near UV	1	SARS-CoV-2	> 4 log	100	Monge et al.^32^

**Figure 5:**
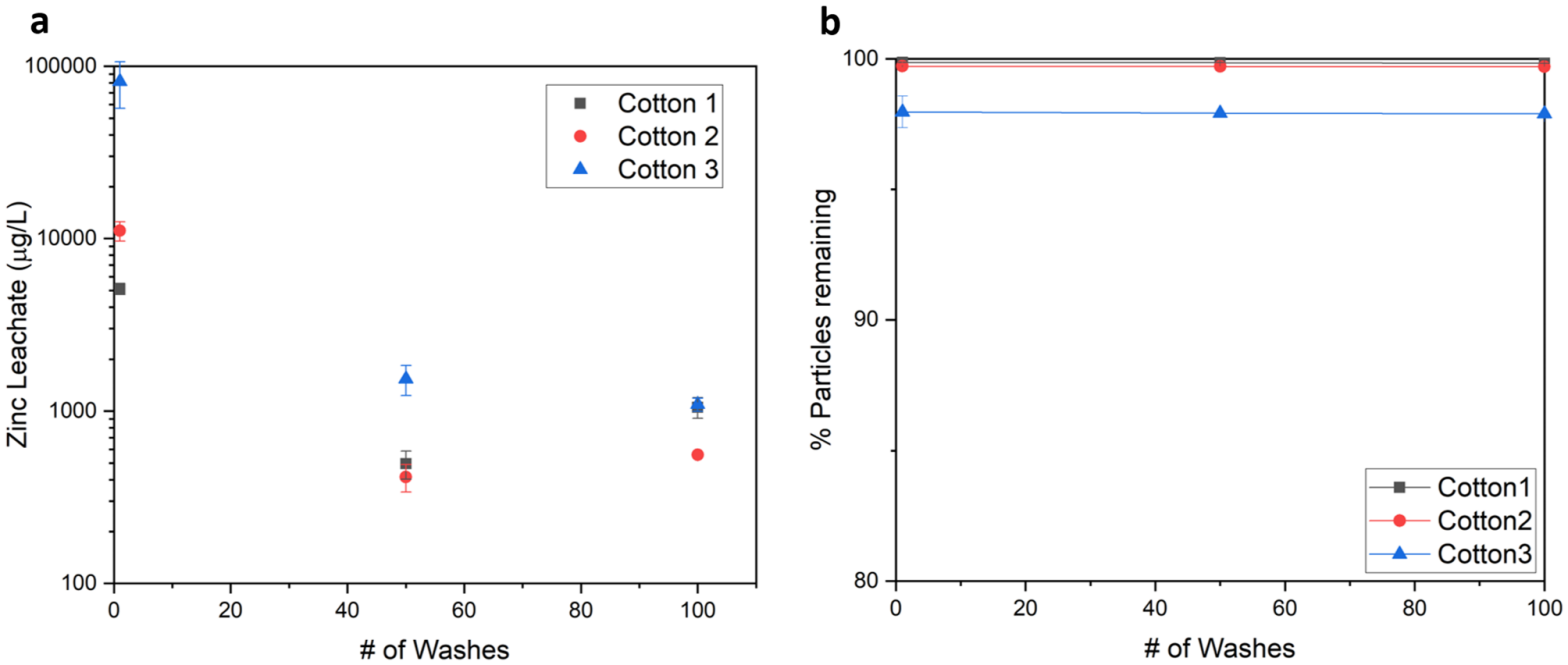
Leaching data of 3 different cotton nanocomposite samples used for consumer facemasks, tested in triplicate. (**a**) Shows data from SPLP tests run by Pace Analytical LLC showing decreased leaching over the life cycle of the product, although the absolute value of zinc leached is below standardized limits for all measurements. (**b**) Is the percentage of zinc particles remaining, calculated by comparing the colorimetric measurement of total zinc at each wash step and the amount lost by SPLP leaching.

Longevity tests were also conducted on various textiles to analyze the level of particle loss that may result from washing the fabric or face masks. Following the post-manufacturing wash where loose particles are removed from the fiber surfaces, sequential washes had insignificant impact on nanoparticle loading even after 100 laundry cycles. Sample fabrics of various cotton types were submitted to Pace Analytical LLC for synthetic precipitation leachate procedure (SPLP) measurements, following EPA 3010A preparation procedure and the EPA 6010B analytical method to measure for zinc concentration^37^. Results from this study showed a maximum zinc leaching of 106,000 µg/L if the fabric is landfilled immediately after production (1 post-fabrication wash), with significantly lower leaching for most samples (see Supplemental Table B). Although there is no federal SPLP limit for zinc, when compared to the soluble threshold limit concentration (STLC) value for the state of California, which is 250,000 µg/L, we can conclude that the amount of zinc leached from our fabric during laundering does not pose a significant hazard^38^. By comparing the SPLP values to active ingredient concentrations obtained via acid digestion and colorimetry measurements (ranging between 10–20 mg/g), we calculate that over 97% of the active ingredient is retained throughout the 100 laundry cycles, indicating remarkably high durability of the antiviral treatment (see Fig. 5). Variations between the different cotton fabrics are likely a result of fiber size and chemical pretreatments, such as mercerization, which will oxidize the fiber surface and make it easier for the metal to bind to the cotton. Cottons 1 and 2 were both mercerized.

The after-wash retention of germicidal properties of our nanocomposite materials was evaluated in a separate work by measuring the antibacterial effect of unwashed, 50 times washed, and 100 times washed samples (see Supplemental Fig. 3). These tests were further validated through third party testing with Vartest Laboratories LLC. Testing was conducted on polyester/cotton blend materials utilizing human coronavirus OC43, a common surrogate for SARS-CoV-2, utilizing the ISO 18184 testing method^39^. These results showed an initial reduction of virus by 99.99% after 0 washes, with the efficacy staying above 99.8% even after 50 laundry cycles. The results demonstrate the ability of the face mask to be reused repeatedly while retaining their properties. Accordingly, we expect retaining the virucidal effect after 100 washes as well. A summary of the antimicrobial test is provided in supplementary information, with third party testing reports available upon request.

### Particle filtration testing

Particle filtration efficiency is a key metric to understand the performance of facemasks, with an increased ability to filter out small particles being indicative of higher protection for the user. Research from hospitals in Wuhan, China demonstrated that the average particle size for viable aerosolized SARS-CoV-2 is between 250 to 500 nm^40^. For maximum protection, healthcare workers are advised to utilize N95 respirators, which can filter out 95% of airborne particles above 300 nm in size^41^. Unfortunately, these respirators are in short supply and are mostly reserved for healthcare workers and first responders, resulting in the general populace utilizing cloth facemasks and surgical masks made from melt-blown polypropylene. Various studies have been conducted to determine the efficacy of different cloth materials and their combinations to improve safety for the average wearer, concluding that three or more layers of cotton or cotton/polyester blends provide the best filtration without impeding breathability^42,43^.

**Figure 6:**
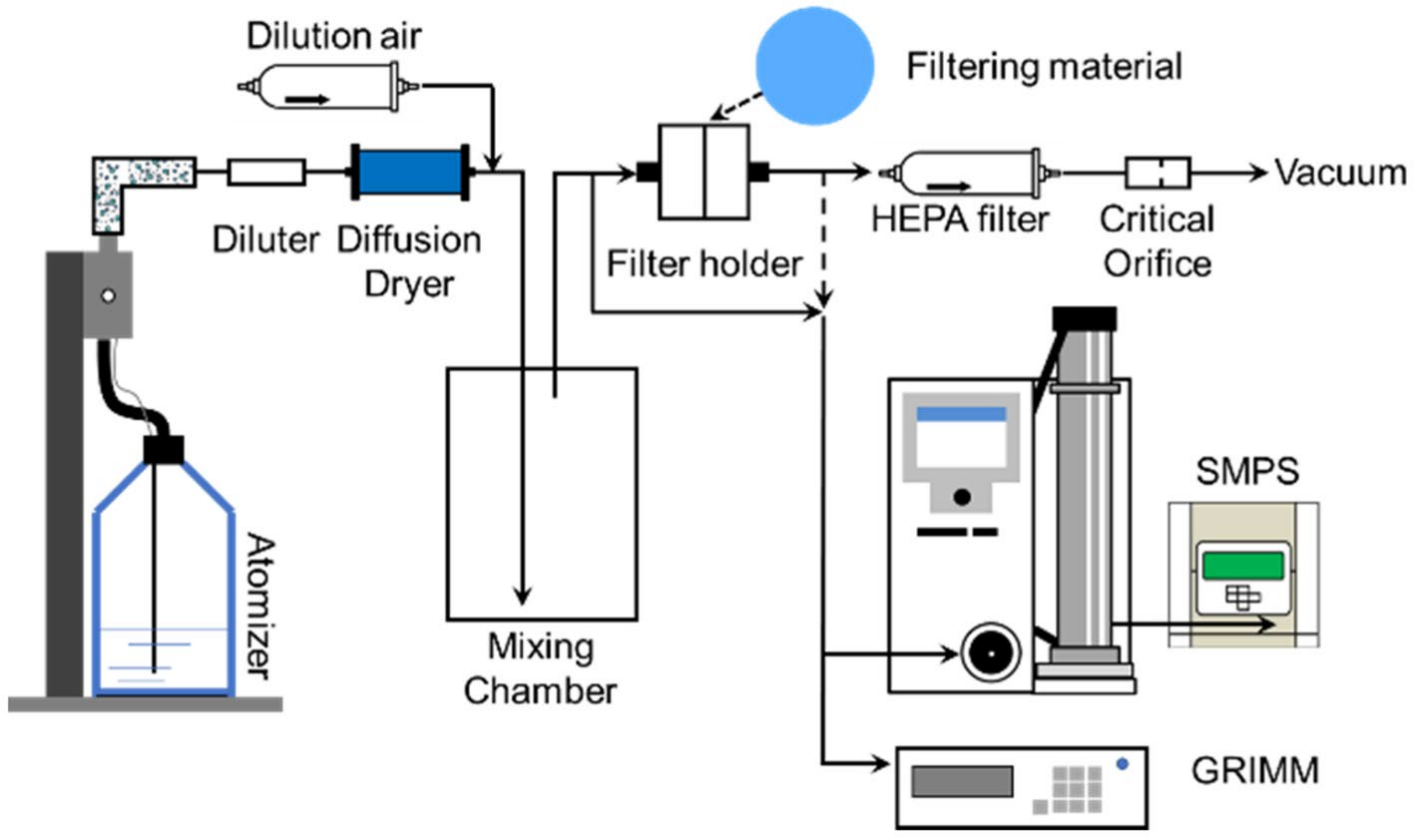
Experimental setup for particle filtration performance testing.

**Figure 7:**
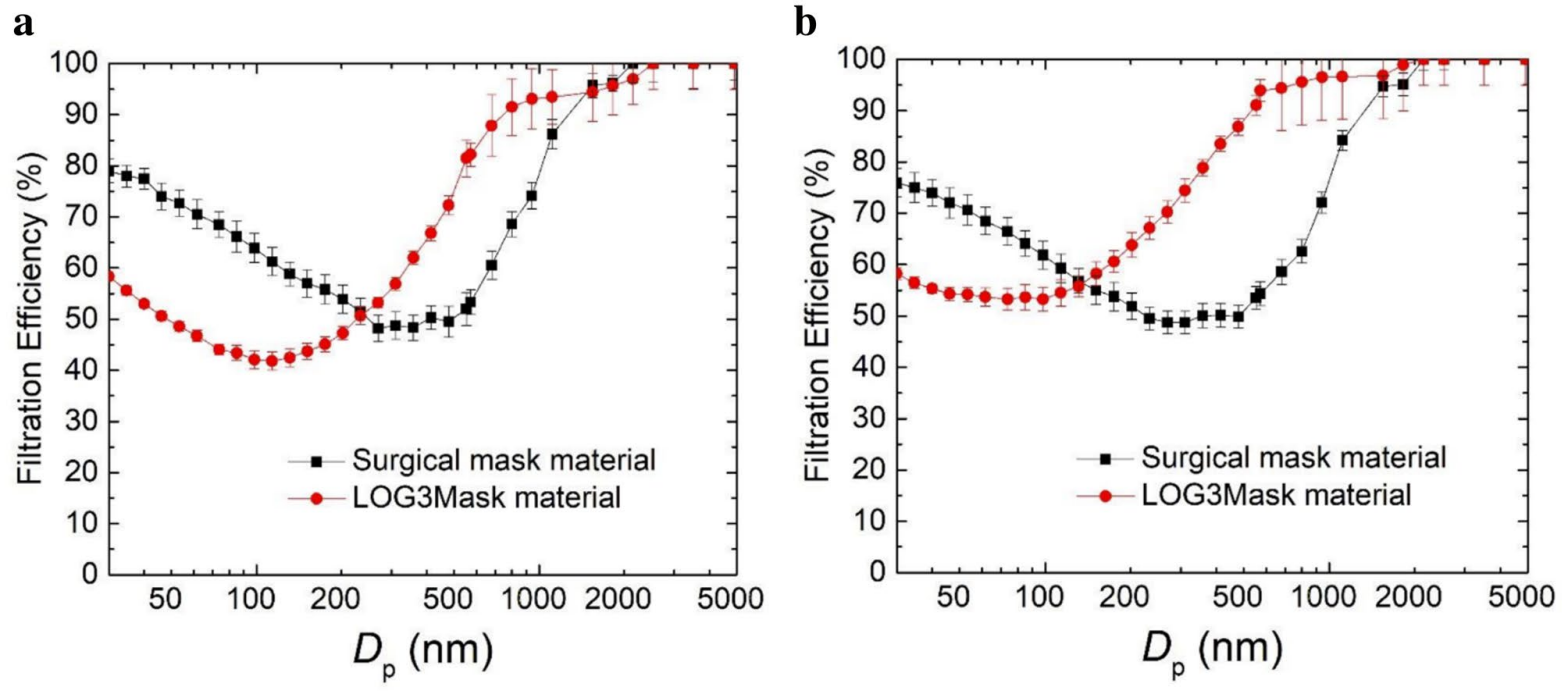
Size-dependent filtration efficiency of the LOG3Mask and a standard surgical mask under a face velocity of (**a**) 15.3 cm s^−1^ and (**b**) 23.2 cm s^−1^.

One variation of the LOG3Mask was developed using cotton fabrics and tested for particulate filtration efficiency at the Particle Measurement and Technology Laboratory at Missouri University of Science and Technology using the setup shown in Fig. 6. Performance was evaluated by measuring the particulate filtration efficiency across a wide spectrum of aerosol sizes (30–5000 nm), as well as the flow resistance through the fabric (breathability) at different face velocities and was compared to a standard surgical mask (3-ply ear loop face mask, Walgreens). Results showed that the filtration efficiency of the LOG3Mask is higher than that of the surgical mask for particle sizes above 200 nm (see Fig. 7a,b), except at a face velocity of 9.2 cm s^−1^ where the filtration efficiency of the LOG3Mask and surgical mask are similar for particle sizes above 500 nm (see Supplemental Fig. 4). The removal of larger particles likely benefited from the enhanced velocity, promoting the impaction and interception of particles on the materials.

Regarding particles with smaller sizes, it is likely that particle residence time is not long enough for particle collection via Brownian motion under the enhanced velocity. The flow resistance shows a higher pressure drop with 0.18 ± 0.03 kPa, compared to 0.09 ± 0.02 kPa for surgical mask materials with the face velocity of 9.2 cm s^−1^. The flow resistance increased with higher face velocities, which are 0.45 ± 0.07, 1.08 ± 0.11 kPa under the face velocities of 15.3, 23.2 cm s^−1^, compared to 0.21 ± 0.02, 0.43 ± 0.03 kPa for surgical mask materials.

## Conclusions

The adaptation of the Crescoating synthesis mechanism to create antiviral nanocomposites for face masks has shown high viral protection with minimal production costs. The zinc oxide material created is safe for use, highly durable, and capable of being washed and dried up to 100 times without loss in functionality. Third party testing conducted by Evalulab Inc., using a Human Repeat Insult Patch Test (HRIPT) method concluded that materials treated with this technology were non-irritating and hypoallergenic. Various designs are also being implemented by Claros Technologies to improve the filtration efficiency compared to the standard surgical mask to offer the best protection for the wearer. The resulting LOG3Mask variations therefore address a growing need for enhanced personal protective equipment that is safe for both human contact and the environment. Further work will be conducted to analyze the efficacy of imparting antiviral functionality on other textiles for healthcare and consumer use, such as lab coats, bed sheets, and even plastics.

## Methods

### Nanocomposite fabrication

Nanocomposites were created by submerging a commercially available face mask textile in a 0.5 M precursor solution of zinc salt in deionized water. The face mask materials tested were melt-blown polypropylene (Vanalay LLC) and 50/50 Cordura^®^ nylon/cotton blend (Rockywoods Fabrics LLC). The hydrophobicity of the polypropylene mask requires that pressure be applied to ensure thorough wetting of the material. The mask, still submerged in solution, was then placed in a commercial convection oven (Model FDL 115, BINDER GmbH, Tuttlingen, Germany) at 100 °C for 4 h. After synthesis, the face masks were subjected to one wash/dry cycle following the American Association of Textile Chemists and Colorists (AATCC) LP1: Home Laundering method in 20 °C water in a machine washer (Vortex M6, SDL Atlas) followed by drying at high temperature in a tumble dryer (Vortex M6D, SDL Atlas). AATCC high efficiency liquid standard reference detergent was used for all wash cycles.

### Zinc colorimetry

Small samples of nanocomposite fabrics between 0.2–0.5 g were digested in an acid mixture of 20 mL nitric acid, 10 mL of 35% hydrochloric acid, and 10 mL of DI water. Digestion is performed by submerging the textile in the acid bath and heating in an oven at 95 °C for 2 h. Analysis of the digested sample is then performed according the USEPA Zincon method 8009 with a Hach DR300 Pocket Colorimeter^44,45^.

### HRIPT testing

The Human Repeat Insult Patch Test (HRIPT) was performed on human participants by Evalulabs LLC to determine skin irritation from a treated fabric. This testing protocol was performed in accordance with relevant guidelines and regulations and was carried out under the direction of a licensed dermatologist. All experimental protocols were approved by an Ethics Committee, the Evalulab LLC Independent Ethics Committee (IEC), to ensure the protection of the rights, safety, and well-being of the subjects participating in the study. Informed consent was obtained from all 50 human subjects and/or their legal guardians. For more details, please see the full report in the supplemental information.

### Virucidal testing

As recommeneded by the ASTM Guidance on SARS-CoV-2 Surrogate Selection, we used TGEV in this testing folllowing the ISO 18184 method with modifications^31,46^. Aliquots (75 µL) of TGEV suspension (with initial titer = ⁓ 6.5 Log TCID_50_/mL) were placed on the center of 2 × 2 cm^2^ sterile parafilm squares that were cut earlier and placed in Petri dishes. Nine squares (2 × 2 cm^2^) of untreated (control) and 9 nanocomposite materials (face mask and nylon/cotton) were placed separately over the surface of each parafilm square where the virus droplets were sandwiched between the tested fabric and the parafilm square. The virus droplets were absorbed immediately by the nylon/cotton specimens as they are hydrophilic while a little pressure by a pipette tip was applied on the polypropylene specimens for sample absorption. After 10 min, 30 min, and 60 min of contact, triplicate sample sets (tested specimen with the absorbed virus and the parafilm square) were withdrawn from the control as well as the nanocomposite specimens. Each sample set was then transferred into a round bottom 13 mL plastic centrifuge tubes (Falcon) containing 1 mL of the virus recovery medium (Eagle’s MEM with 4% FBS and standard antibiotics). All tubes were then vortexed for 2 min immediately after transferring to recover the viral particles from the tested specimens. In a concurrent experiment (leached NP control), nanocomposite specimens (unspiked with the virus) were transferred first into virus recovery tubes and vortexed for 2 min followed by the addition of 75 µL aliquot of the virus into each tube (without direct contact with the fabric). This was done to know whether a fraction of viral particles was inactivated by contact with nanoparticles that might have leached in the virus recovery solution following the recovery of the virus from the fabric.

The titer of surviving virus recovered in the recovery medium was performed by the 50% tissue culture infective dose (TCID_50_) method. Serial tenfold dilutions were prepared from the recovery medium of each sample. These dilutions were inoculated in 80% confluent monolayers of swine testicular (ST) cells, pre-prepared in 96-well microtiter plates using 3 wells per dilution (100 μL of each sample dilution/well). The infected cells were incubated at 37 °C in a 5% CO_2_-incubator for up to five days and examined daily under an inverted microscope for the appearance of cytopathic effects (CPE). The highest dilution of the virus, which produced CPE in 50% of the infected cells, was considered as the endpoint. The titer of the surviving virus in each sample was then calculated by the Karber method and expressed as log_10_ TCID_50_/sample^47^.

### Real time RT-qPCR

To gain some insights on the mode of action of virus inactivation by the nanocomposites, we quantified the viral genome copy numbers in the recovery solution after virus recovery from the control and nanocomposite specimens. Viral RNA was extracted from 140 μL of each sample using QIAamp DSP Viral RNA Mini Kit (Qiagen, Germany) according to the manufacturer’s instructions. The RNA was eluted in 100 μL of elution buffer and stored at − 80 °C until used for viral genome quantification. For RT-qPCR, we used PCR primer set and probe shown in Table 2. The RT-qPCR primers were designed to target a conserved 146 bp region (corresponding to the region between nucleotides 370 and 515 of the TGEV S gene with reference to the sequence of TGEV-GenBank accession no.: KX900410.1). The primers and probe were manufactured by Integrated DNA Technologies (IDT, Coralville, IA). The RT-qPCR reactions were performed using AgPath-ID One-Step RT-PCR kit (Applied Biosystems by Thermo Fisher Scientific, Waltham, MA). The reaction mixture (25 μL) consisted of 5 μL of template RNA, 12.5 μL of 2 × RT-PCR buffer, 1 μL 25 × RT-PCR Enzyme Mix, 0.50 μL of 10 μM forward primer (200 nM final concentration), 0.50 μL of 10 μM reverse primer solution (200 nM final concentration), 0.30 μL of 10 μM probe (120 nM final concentration), and 5.20 μL of nuclease-free water. The RT-qPCR was performed in the QuantStudio™ 5 PCR thermocycler system (Thermo Fisher Scientific, Applied Biosystems™). Reverse transcription was performed at 45 °C for 10 min. Taq polymerase activation was done at 95 °C for 15 min followed by 45 amplification cycles using a 95 °C/15 s denaturation step and an annealing/extension step at 58 °C for 45 s. Fluorescence was measured at the end of annealing step in each cycle. In each run of RT-qPCR, standard curve samples and no template control were used as positive and negative controls, respectively. The TGEV standard/calibration curve was constructed for absolute quantification of viral genome copy number, in which we used serial ten-fold dilutions of a 557 bp purified conventional RT-PCR amplicon of TGEV S gene (including the 146 bp target sequence of the RT-qPCR prime/probe set). The 557 bp TGEV S gene fragment was produced by conventional RT-PCR reaction using an in-house developed primer set shown in Table 2. Results were expressed as cycle threshold (Ct) values. The Ct values and standard curve were used to calculate the absolute genome copy number of TGEV, expressed as genome copies/sample.

**Table 2:** Oligonucleotides for TaqMan-based TGEV RT-qPCR used in this study.

Name of PCR reaction	Oligo-nucleotide name	Sequence (5′–3′)	Polarity^a^	Position	Product length (bp)	References
TGEV RT-qPCR	TGEV-F	TCTGCTGAAGGTGCTATTATATGC	+	20,722–20,745^c^	146	^44^
	TGEV-R	CCACAATTTGCCTCTGAATTAGAAG	−	20,867–20,843^c^		
	TGEV-P	FAM-TAAGGGCTC/ZEN/ACCACCTACTACCACCA-3IABkFQ	+	20,751–20,776^c^		
TGEV RT-PCR (for standard curve construction)	TP-F	GCAGGTTACCACCTAATTCAGA	+	20,486–20,507^c^	557	H. Aboubakr using primer 3 plus
	TP-R	CAGGATTAAACCACCAAAGGTC	−	21,043–21,022^c^		

^a^+, virus sense; −, anti-virus sense.

^c^Corresponding nucleotide position of TGEV genome (GenBank accession no.: KX900410.1) as reference.

### Statistical analysis

The virucidal testing experiment was performed twice. In each of them, triplicate samples were tested at each contact time. Hence, the results presented here are the geometric means of 6 replicates ± one gemoetric standard diviation. The presented results of real-time RT-PCR are the geometric means of dublicate samples ± one gemoetric standard diviation. The analysis of variance was pefromed by One-way ANOVA and the significance of differences between the means were performed by paired comparisons using Tukey’s test at significance = 0.05.

### Germicidal testing

The germicidal efficacy can be determined through standardized American Association of Textile Colorists and Chemists (AATCC) methods that evaluate the antibacterial effects and determine the minimum inhibitory concentrations (MICs) and minimum bactericidal concentration (MBCs) of the different metal nanoparticles on the select microorganisms. Quantitative and qualitative tests based on standardized AATCC TM100-2004 and AATCC TM147-2004 protocols were utilized, respectively^48^. Per these methods, *Staphylococcus aureus *subsp*. aureus* (ATCC 6538) were grown in tryptic soy broth (TSB) to get 10^8^ cells. This was followed by serial dilution using TSB to get a final concentration of 10^5^ for inoculation purposes. Two swatch sets of untreated fabric and one swatch set of treated fabric were each placed in a 60 mm × 15 mm petri dish. The swatches were separately inoculated with 1 mL of the bacterial strain in broth prepared as described above. Broth solutions were ensured to completely soak into each fabric. A control swatch was inoculated with 1 mL of sterile TSB as a measure of contamination. These petri dishes were sealed with paraffin plastic film and incubated for 24 h at 37 °C. For immediate elution tests, inoculated swatch samples were prepared as described above and were each transferred to tubes containing 50 mL of 0.15 M NaCl solution. The swatches were compressed against the walls of the tube and vortexed to ensure complete elution of the inoculum. After thorough elution, the swatches were removed from NaCl solution. A separate tube containing 50 mL of NaCl solution was directly inoculated with bacterial culture to serve as a control. These elution steps were repeated for the 24-h incubated swatch samples. The eluate for each sample was serially diluted once using TSB for the immediate elution samples and five times for the 24 h incubated swatch samples before plating on tryptic soy agar (TSA) and incubating for 24 h at 37 °C. After incubation, bacterial colonies were counted, and percent reduction was calculated as described in the following section.

The antimicrobial activity of nanocomposite fabrics was tested against *Staphylococcus aureus* (Gram-positive bacteria) as outlined in the AATCC methods. The microorganism *Staphylococcus aureus* is a pathogenic bacterium and was manipulated in Biosafety Laboratory Level 2 (BSL2) at the Veterinary Diagnostic Laboratory at the University of Minnesota. Quantitative methods are based on calculating the reduction percent of bacteria from 0 to 24 h contact time from both the inoculated fabric sample and control sample (with no antibacterial agent). The percentage reduction is determined as follows:

$$ R\;(\% ) = \frac{A - B}{A} $$

where, R is the reduction in colony forming units (cfu), A is the number of bacterial colonies from the control textile, and B is the number of bacterial colonies from the treated textile. The qualitative assessment of antibacterial activity was performed using a parallel streak method (AATCC 147-2004), and the quantitative test was performed using AATCC 100-2004 method.

### Filtration performance testing

The experimental setup is shown in Fig. 5. The test aerosols were produced from a NaCl-water solution with a mass concentration of 0.1% by using a constant output atomizer (Model 3076, TSI Inc., Shoreview, MN). The aerosols were diluted, dried, and then homogenized in a mixing chamber. The LOG3Mask material was cut into a disc with a diameter of 37 mm and tightly pressed onto the mesh support of a filter cassette (Air Sampling Cassette, Zefon International Inc., Ocala, FL) and sealed at the edge. The size distributions of aerosols in the range of 30–600 nm upstream and downstream of the filter holder and the concentration of the mobility-classified particles were determined by a scanning mobility particle sizer (SMPS, Model 3936, TSI Inc., Shoreview MN). A portable aerosol spectrometer (GRIMM Model 11-D, Durag Inc., Mendota Heights, MN) measures the size distributions of aerosols in the 500–5000 nm range. A two-digit manometer (RISEPRO, 365BG947677, measuring range ± 13.79 kPa, 0.001 kPa resolution) was used to track the flow resistance of the materials, as the flow resistance across the filter material is a crucial component for determining the breathability of the material. The filtration efficiencies obtained from the size distributions measured by the SMPS and GRIMM, and the size-dependent filtration efficiency ($\eta (D_{p} )$) was calculated by:

$$ \eta (D_{p} ) = 1 - \frac{{n_{o} (D_{p} )}}{{n_{i} (D_{p} )}} $$

where $n_{o} (D_{p} )$ and $n_{i} (D_{p} )$ are the particle number concentrations for each particle size measured at the outlet (downstream) and inlet (upstream) of the filter cassette. The standard deviation was calculated based on the error propagation method discussed in Hao et al.^49,50^.

## Supplementary Information

Supplementary Information.
