# Supplementary material for: Durable nanocomposite face masks with high particulate filtration and rapid inactivation of coronaviruses
Source: Sci Rep. 2021 Dec 21;11:24318. doi: 10.1038/s41598-021-03771-1 (PMC8692499; doi:10.1038/s41598-021-03771-1)

Durable Nanocomposite Face Masks with High Particulate Filtration and Rapid Inactivation of Coronaviruses

*Andrew Gonzalez^1^, Hamada A. Aboubakr^2^, John Brockgreitens^1^, Weixing Hao^3^, Yang Wang^3^, Sagar M. Goyal^2^, Abdennour Abbas^1,4^**

^1^ Claros Technologies Inc.

1000 Westgate Drive

Suite 1005

St. Paul, MN 55114, USA

^2^ Department of Veterinary Population Medicine,

University of Minnesota Twin Cities

1333 Gortner Ave.

St. Paul, MN 55108, USA

^3^ Department of Civil, Architectural, and Environmental Engineering

Missouri University of Science and Technology

1401 N Pine St.

Rolla, MO 65409, USA

^4^ Department of Bioproducts and Biosystems Engineering

University of Minnesota-Twin Cities

2004 Folwell Ave

St. Paul, MN 55108, USA

A.E.G and H.A.A. contributed equally to this study

Corresponding author: Dr. Abdennour Abbas

Email: [aabbas@umn.edu](mailto:aabbas@umn.edu), dennor@clarostech.com

Phone #: +1 (612) 624-4292

**Supplementary Information**


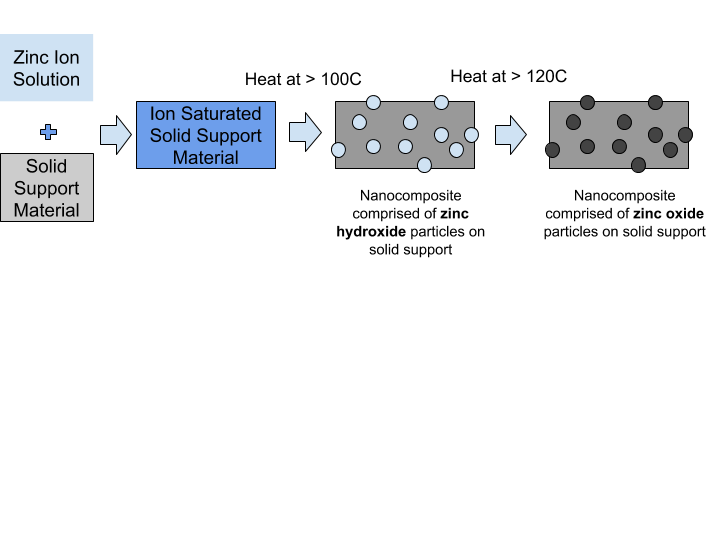


**Step 1. Hydrolysis:**

**
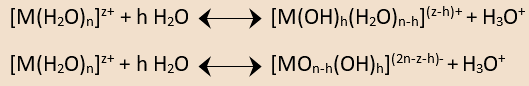
**

**Step 2. Hydroxo bridging–oxolation:**

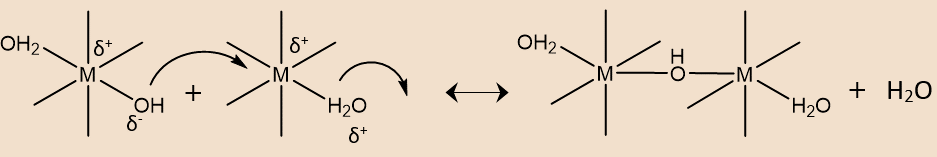


**Step 3. Oxo bridging–oxolation:**

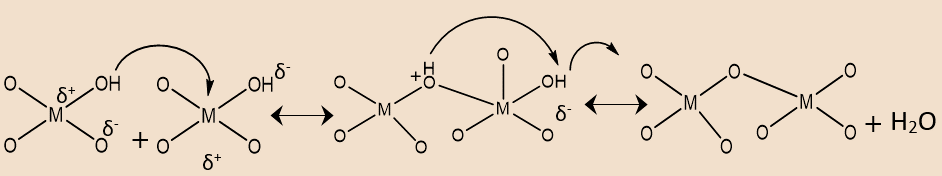


**Supplementary Figure 1:** Schematic of the zinc oxide formation process, starting from submersion of the textile in a precursor solution with M denoting the metal atom. The textile undergoes two changes under heat, the first being a hydrothermal growth process during which hydroxide intermediates are formed (Step 1) from the initial metal salt, followed by decomposition of zinc hydroxides into zinc oxide at higher temperatures by oxolation (Step 2 and 3).


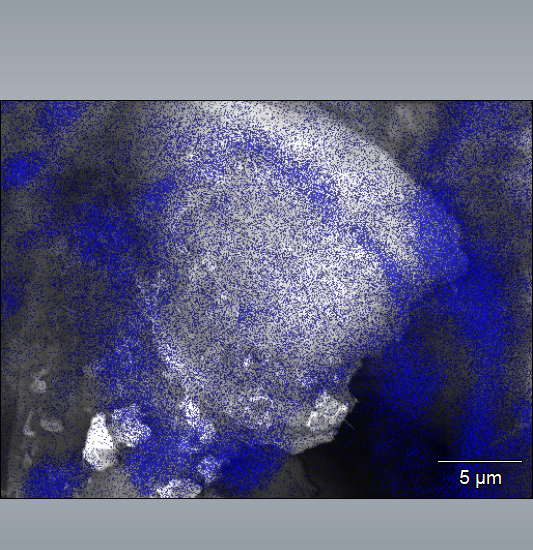


**B**

**A**


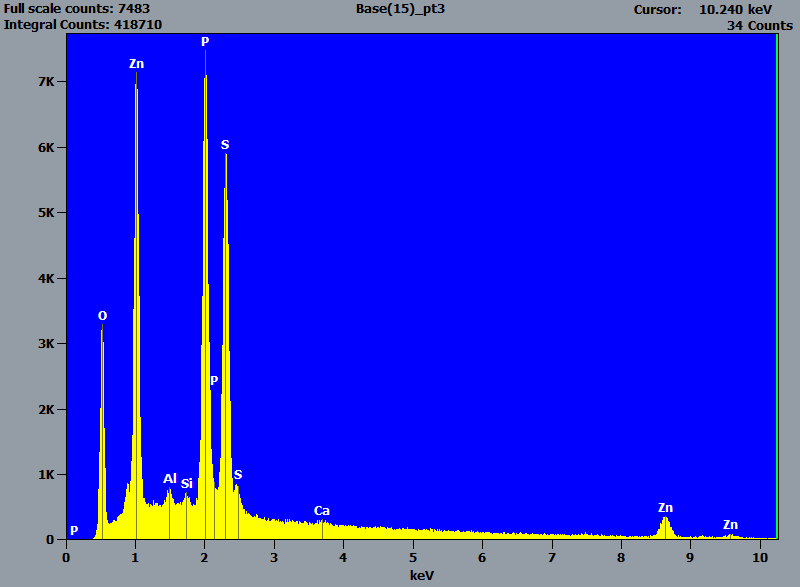

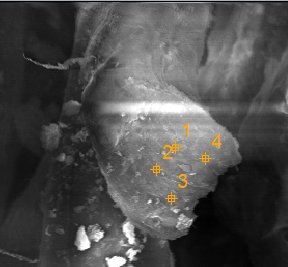


**Supplementary Figure 2:** EDX images of a nylon/cotton fabric cross-section showing the elemental analysis of the fiber (**A**) and an overlay of the SEM image with Zinc signal (**B**). The high levels of zinc present demonstrate the formation of a nanocomposite where nanoparticles have been distributed throughout the bulk of the fiber and not simply deposited on the surface.

**Supplementary Table A:** Loading of zinc nanoparticles in the bulk and surface of the nylon/cotton textile fibers.

|  | Atomic weight (%) | Atoms (%) |
| --- | --- | --- |
| Zinc nanoparticles grown inside the textile fibers | 27 ± 3 | 12 ± 2 |
| Zinc nanoparticles grown on the surface of the textile fibers | 42 ± 2 | 20 ± 4 |

Analysis of the EDX measurements reveals higher levels of zinc nanoparticles on the surface compared to within the bulk of the material.

**Supplementary Table B:** Synthetic Precipitate Leachate Procedure results from Pace Analytical from three different cotton samples obtained from an industry partner. Each sample was washed 1, 50, and 100 times from the point of functionalization.

| **Zinc Discharge (µg/L)** | | | | | | | | | |
| --- | --- | --- | --- | --- | --- | --- | --- | --- | --- |
|  | # of Washes | | | | | | | | |
|  | **1** | | | **50** | | | **100** | | |
| **Cot #1** | 5030 | 5610 | 4680 | 543 | 575 | 366 | 1150 | 848 | 1160 |
| **Cot #2** | 12800 | 11100 | 9350 | 519 | 371 | 352 | 571 | 558 | 544 |
| **Cot #3** | 57100 | 106000 | N/A | 1500 | 1180 | 1930 | 1180 | 1140 | 961 |

We observe that most of the Zinc leaching occurs after the first wash, likely when larger, loosely bound surface particles are easily washed away. After this initial wash, there is significantly lower leaching throughout the life of the fabric.

**
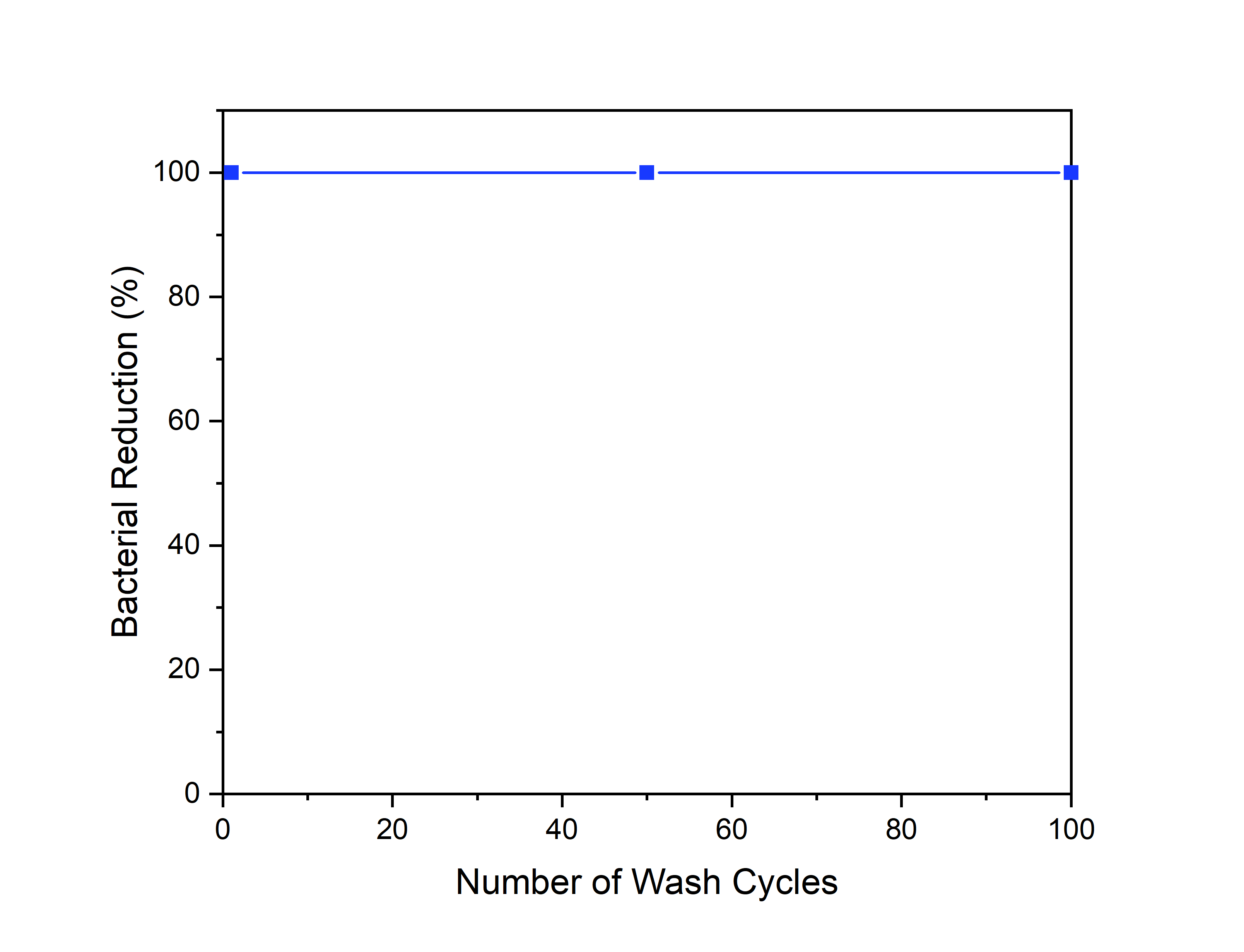
**

**Supplementary Figure 3:** Bacterial reduction of gram-positive bacteria *Staphylococcus aureus* using a modified AATCC 100 method on a treated cotton nanocomposite. The results indicate that even after 100 washes the antibacterial efficacy remains unaffected due to the growth mechanism of the nanoparticles.


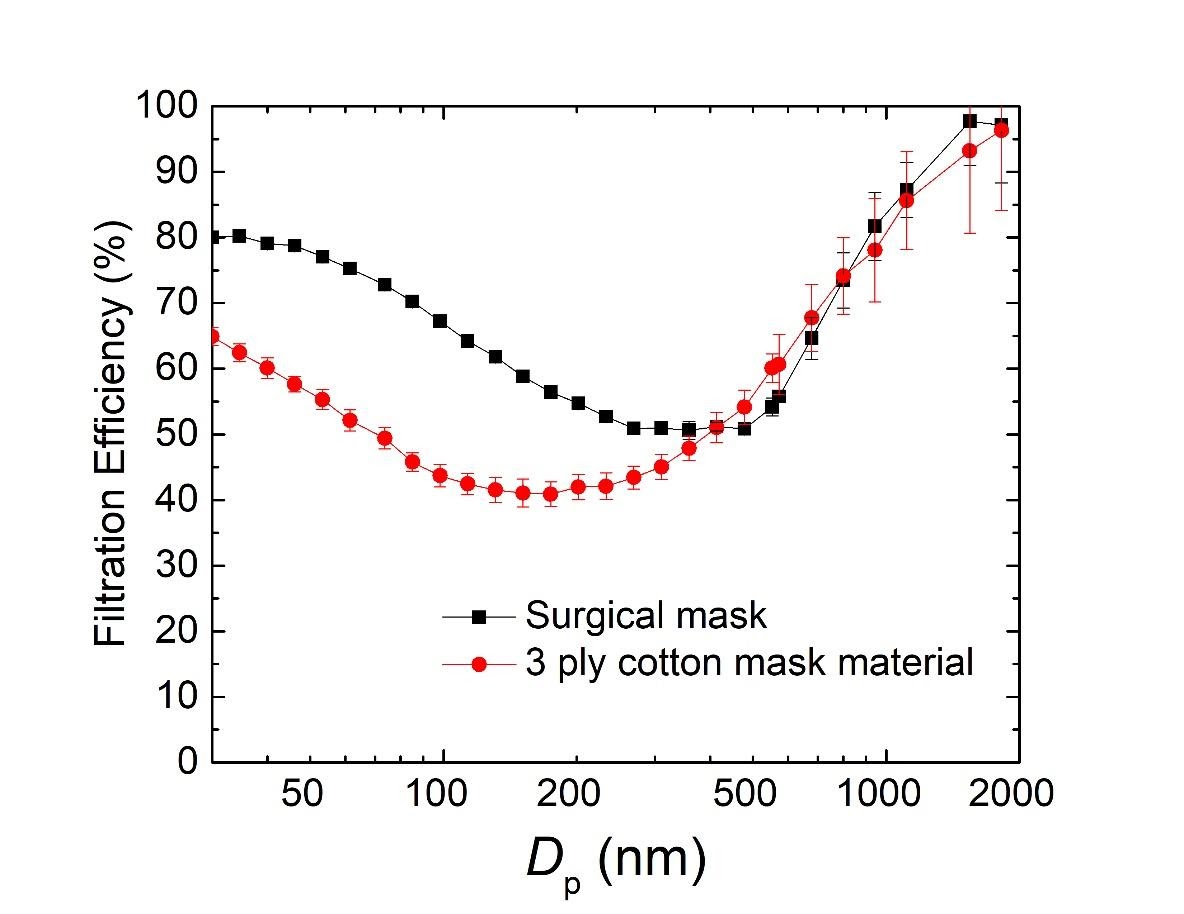


**Supplementary Figure 4:** Size-dependent filtration of LOG3Mask and standard surgical mask under a face velocity of 9.2 cm s^-1^. Filtration efficiency is similar for particles larger than 500nm, however for particles with smaller sizes, it is likely that particle residence time is not long enough for particle collection via Brownian motion under the provided velocity.


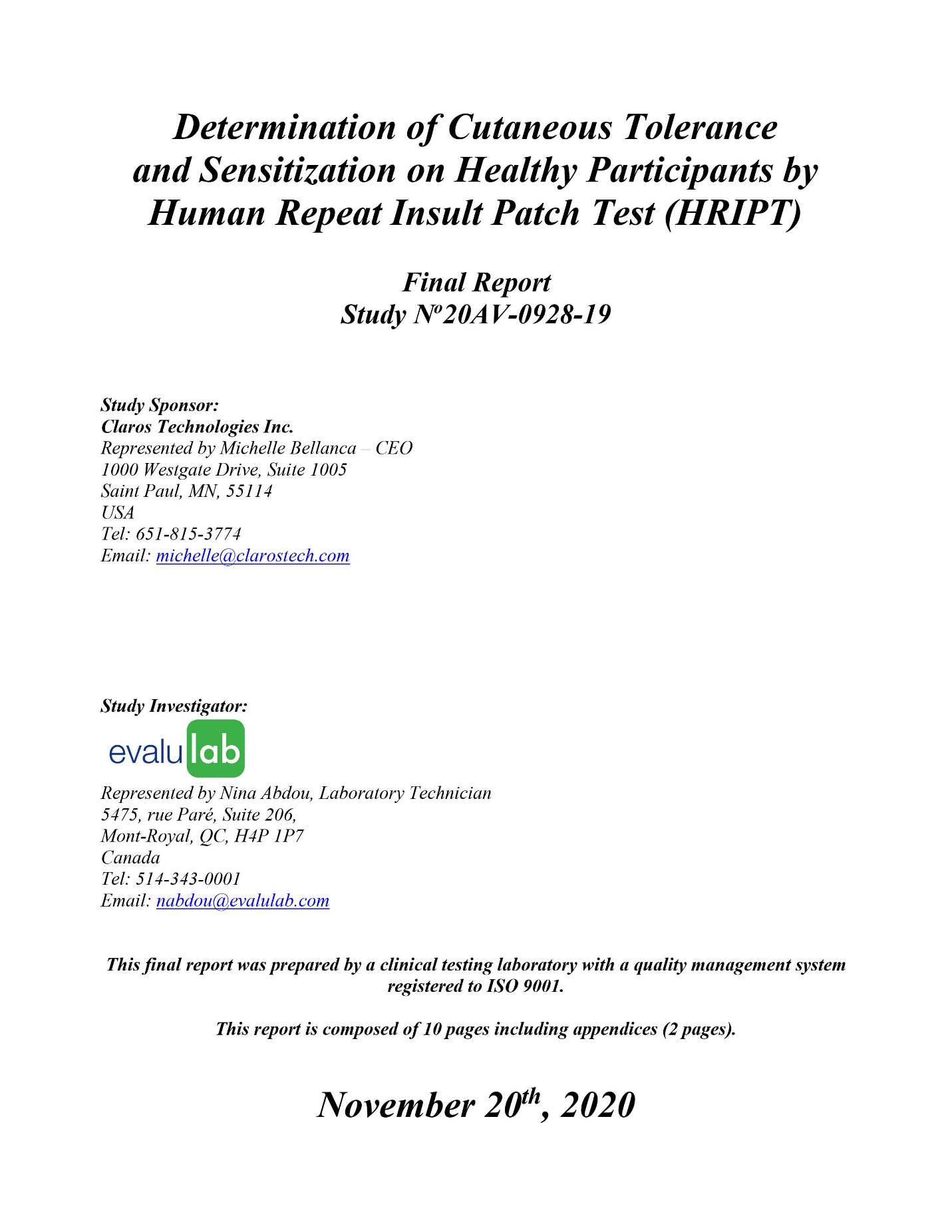


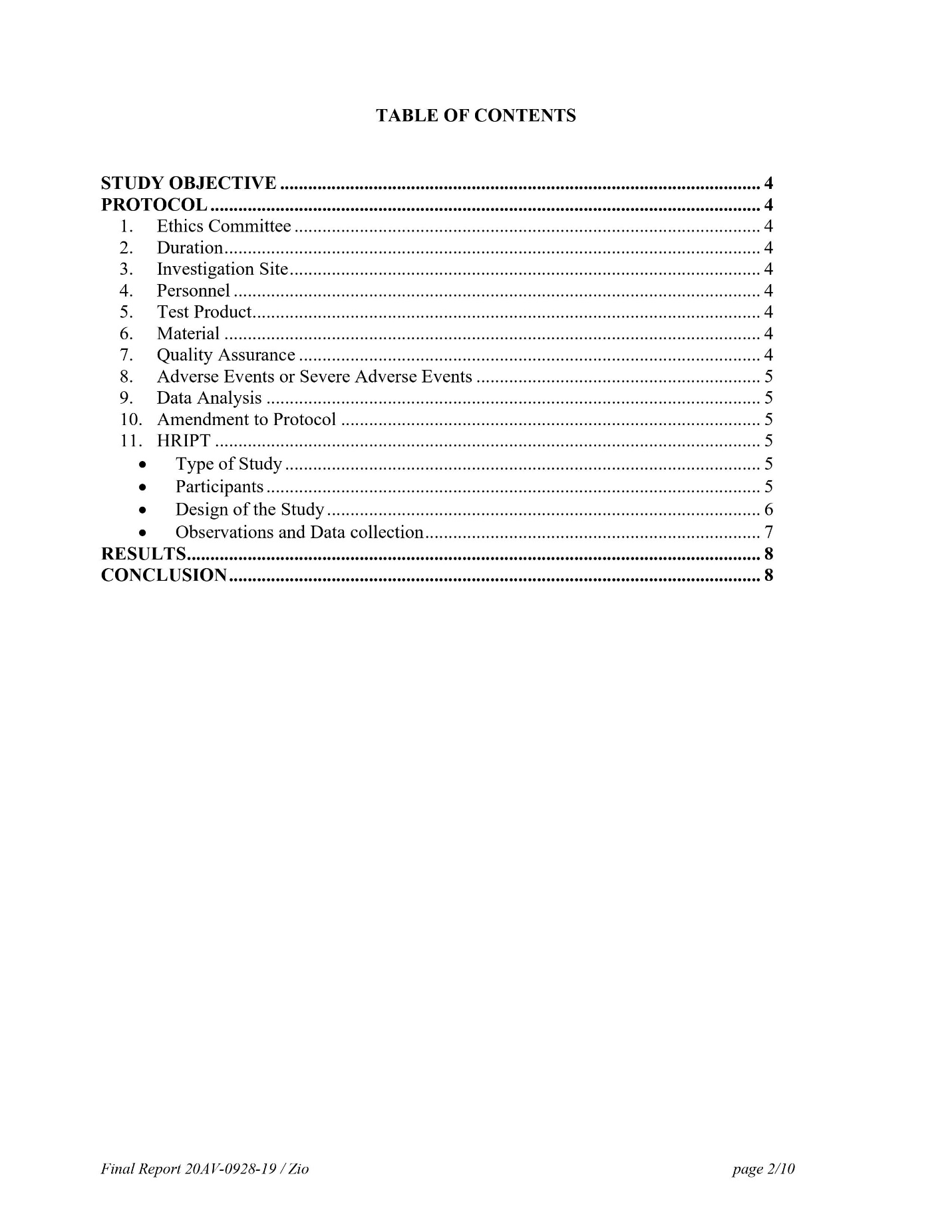


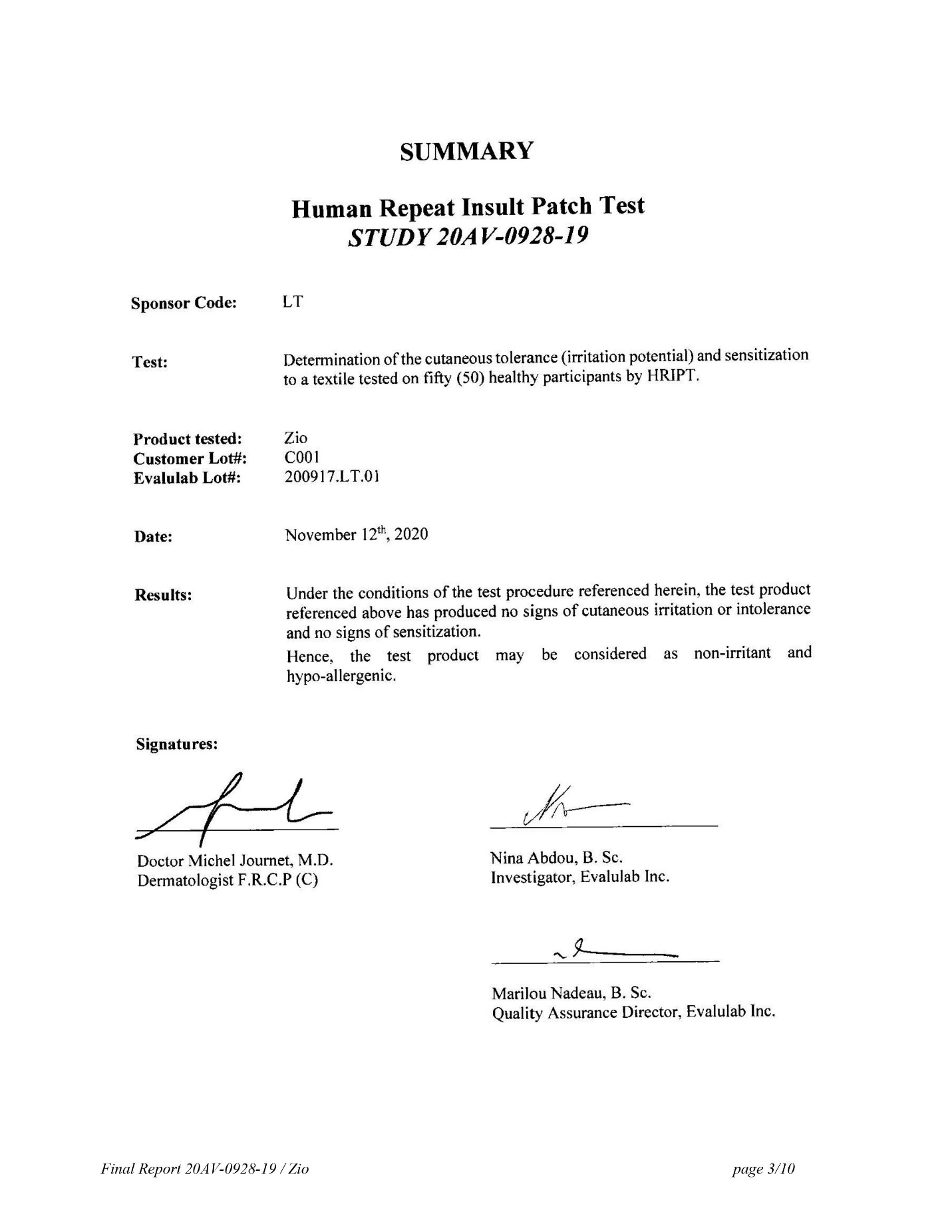


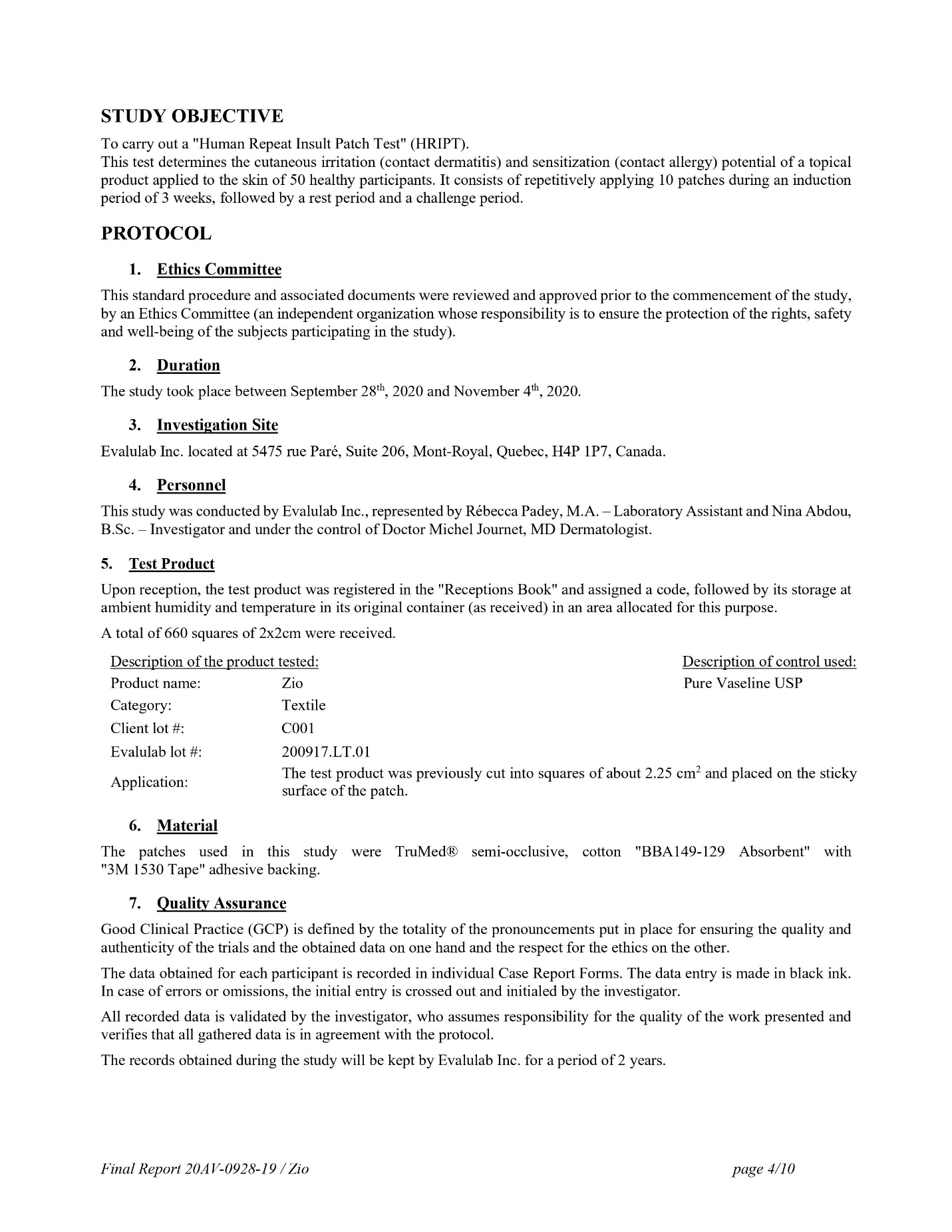


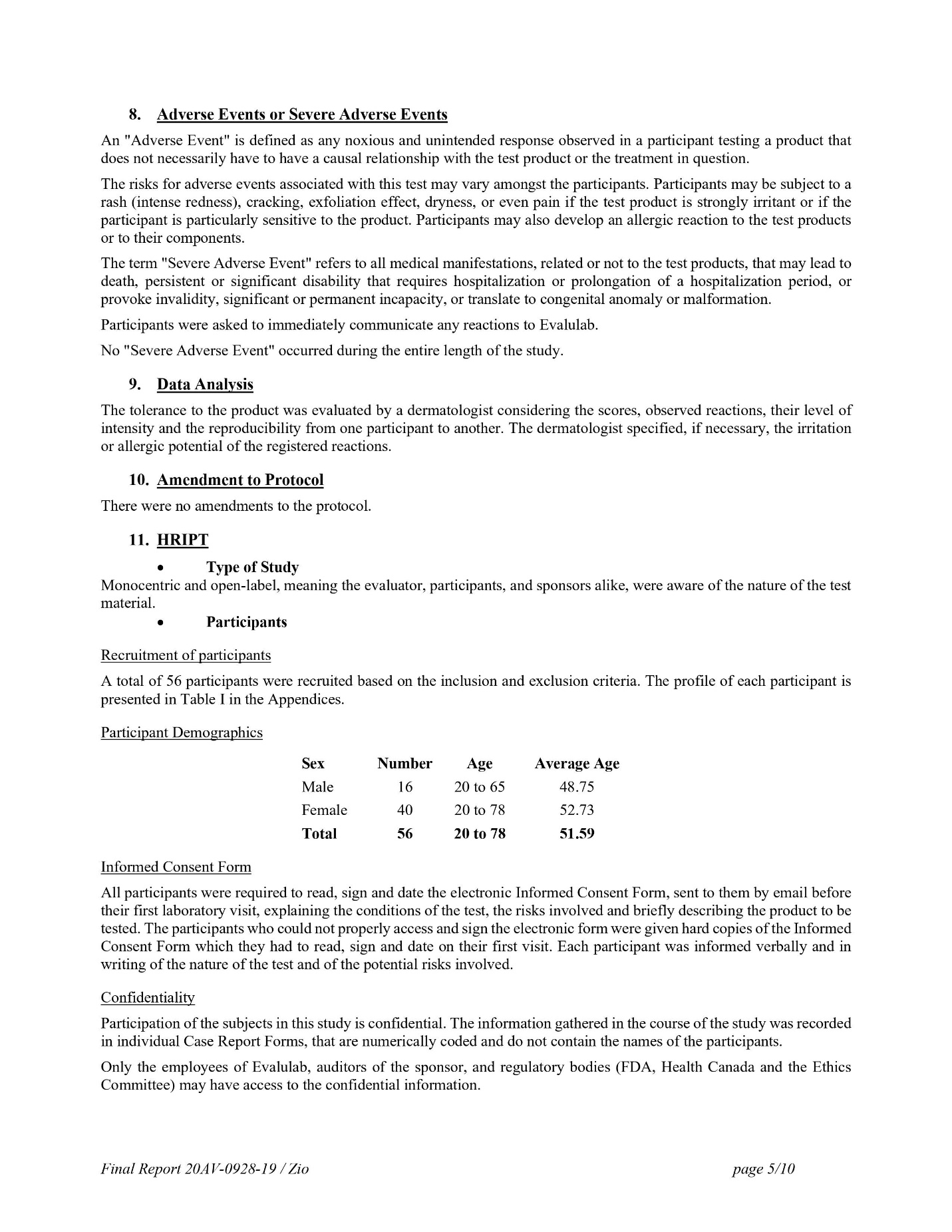


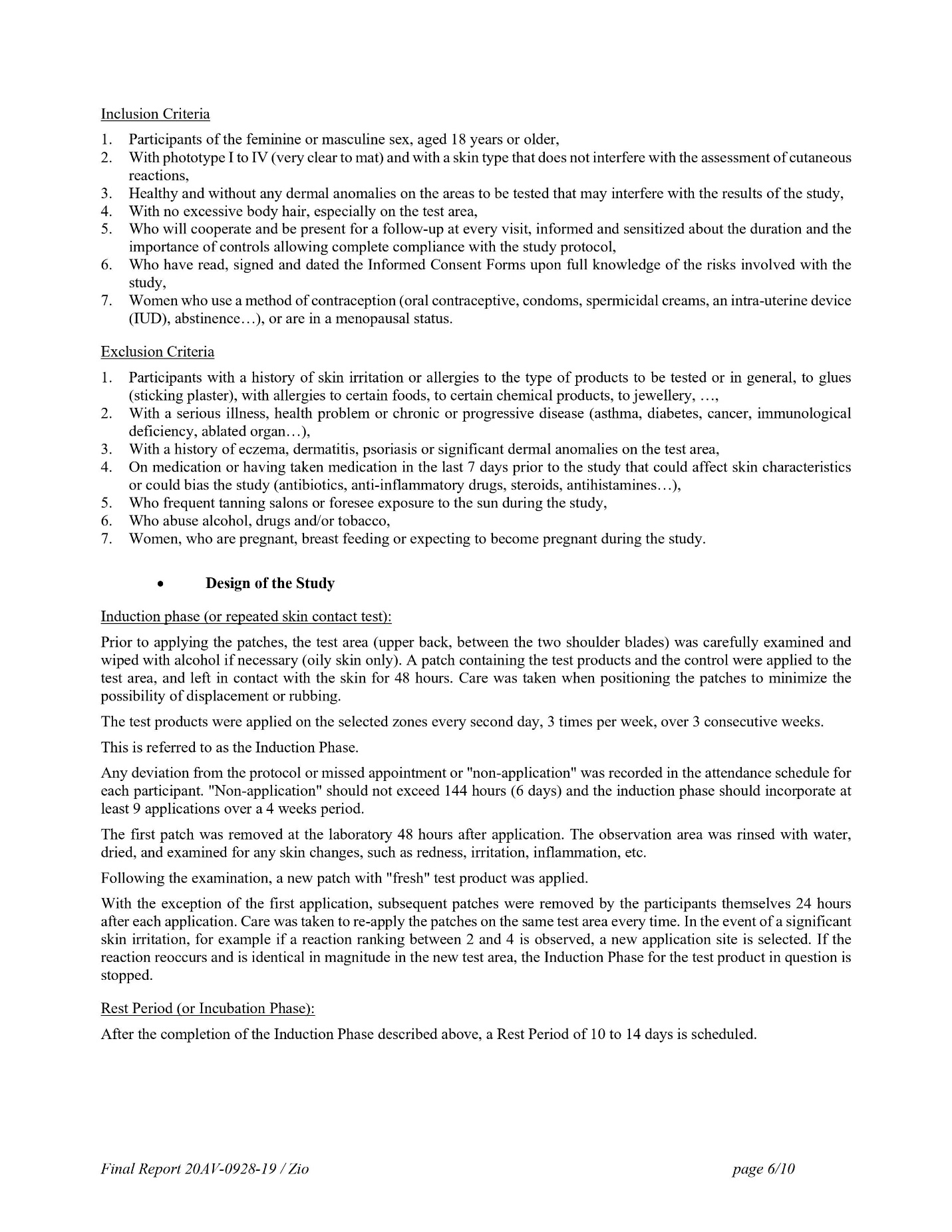


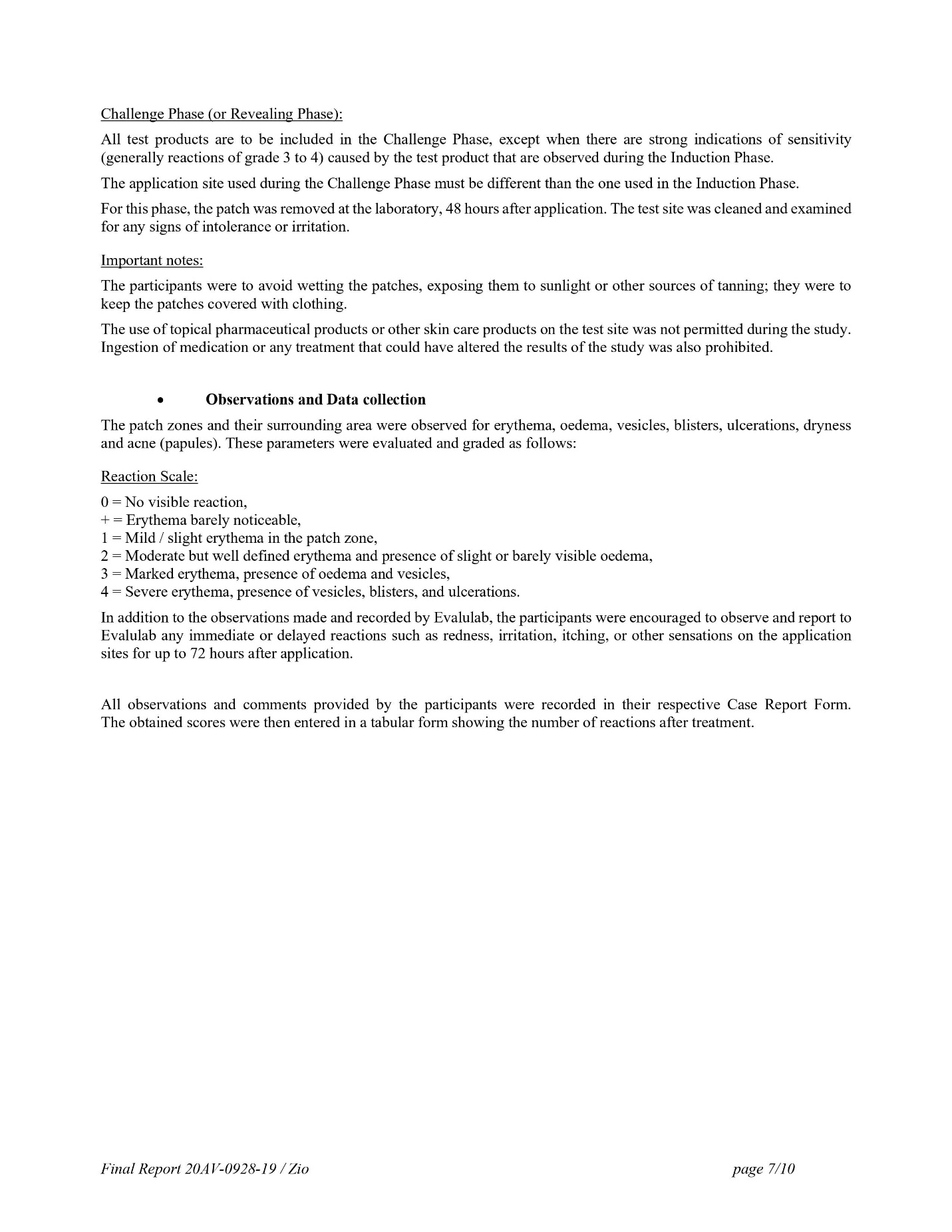


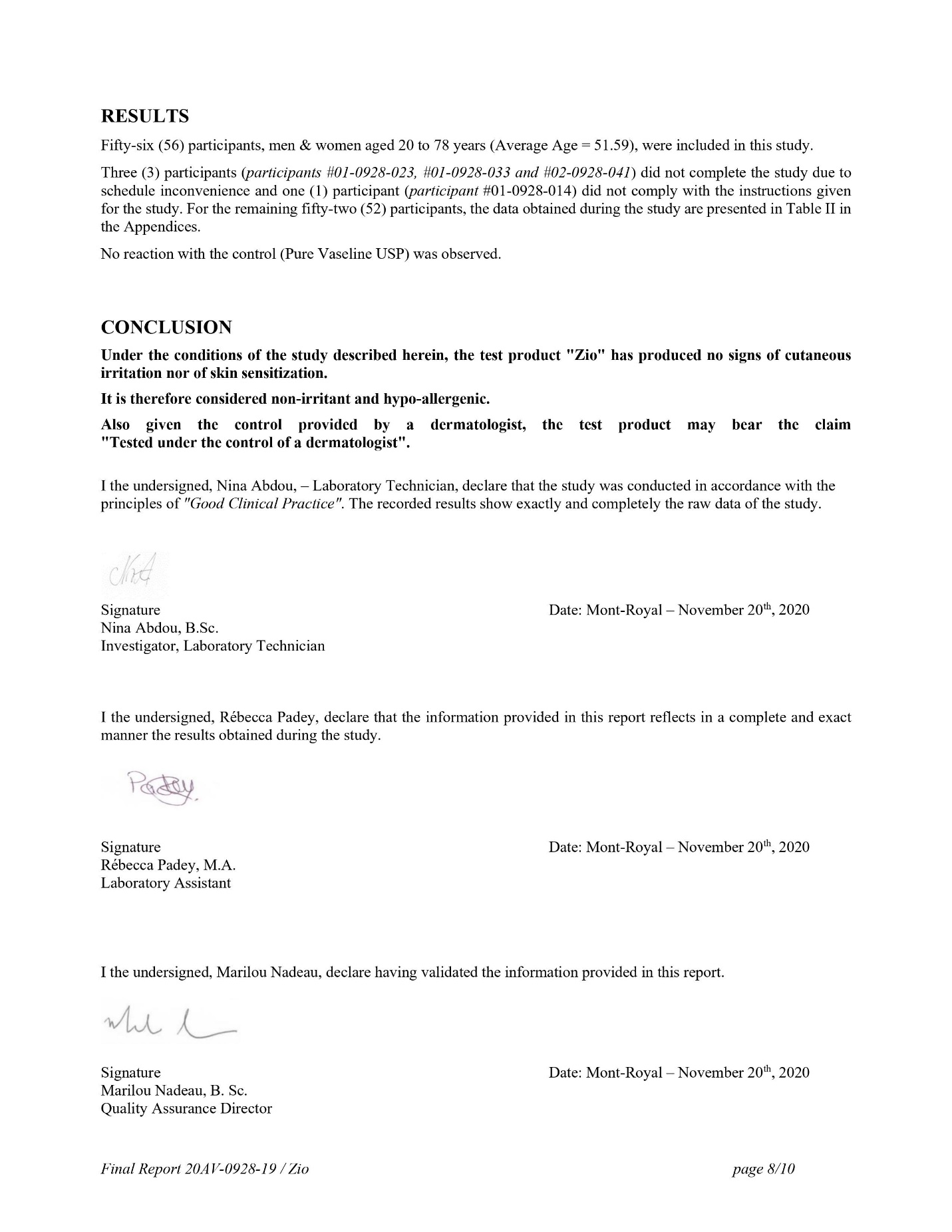


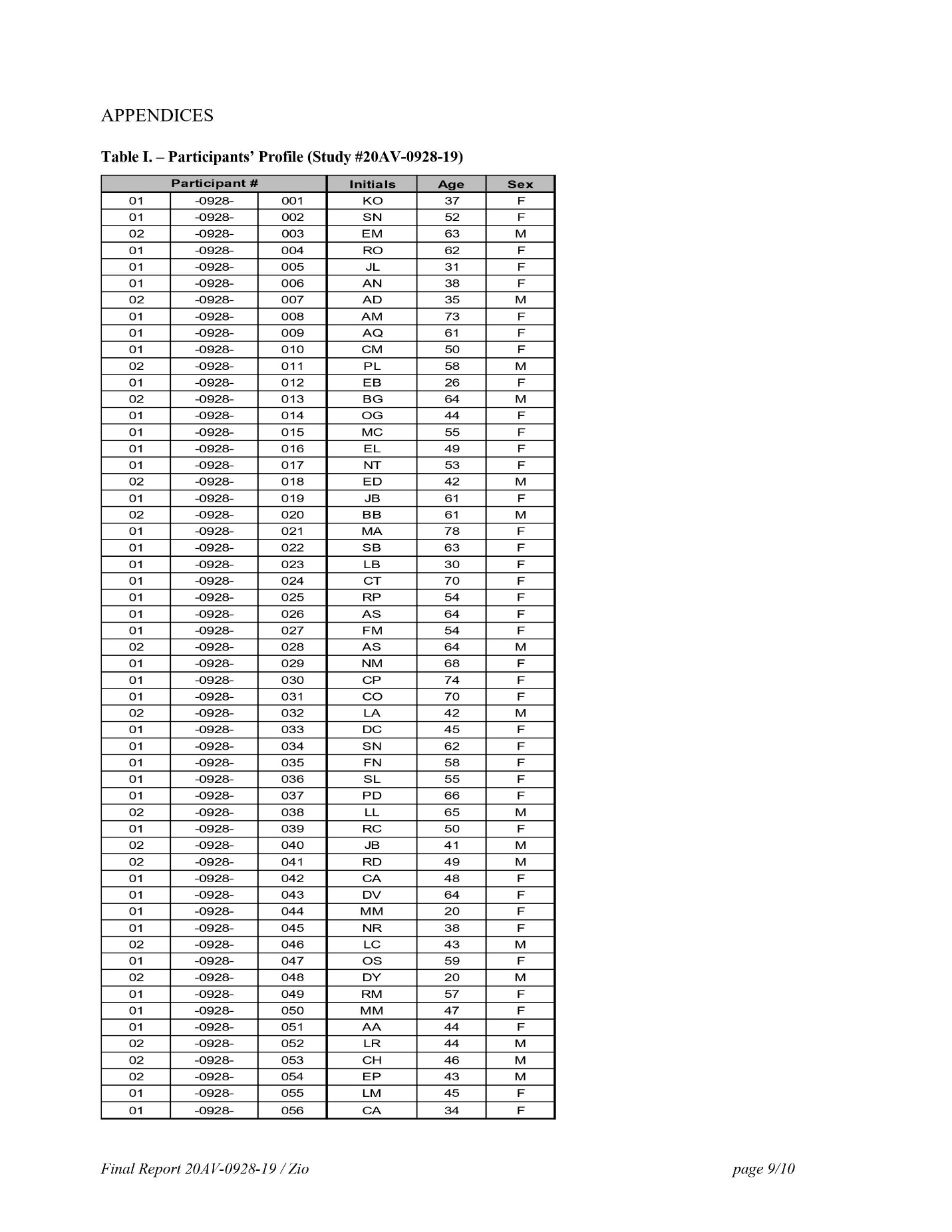


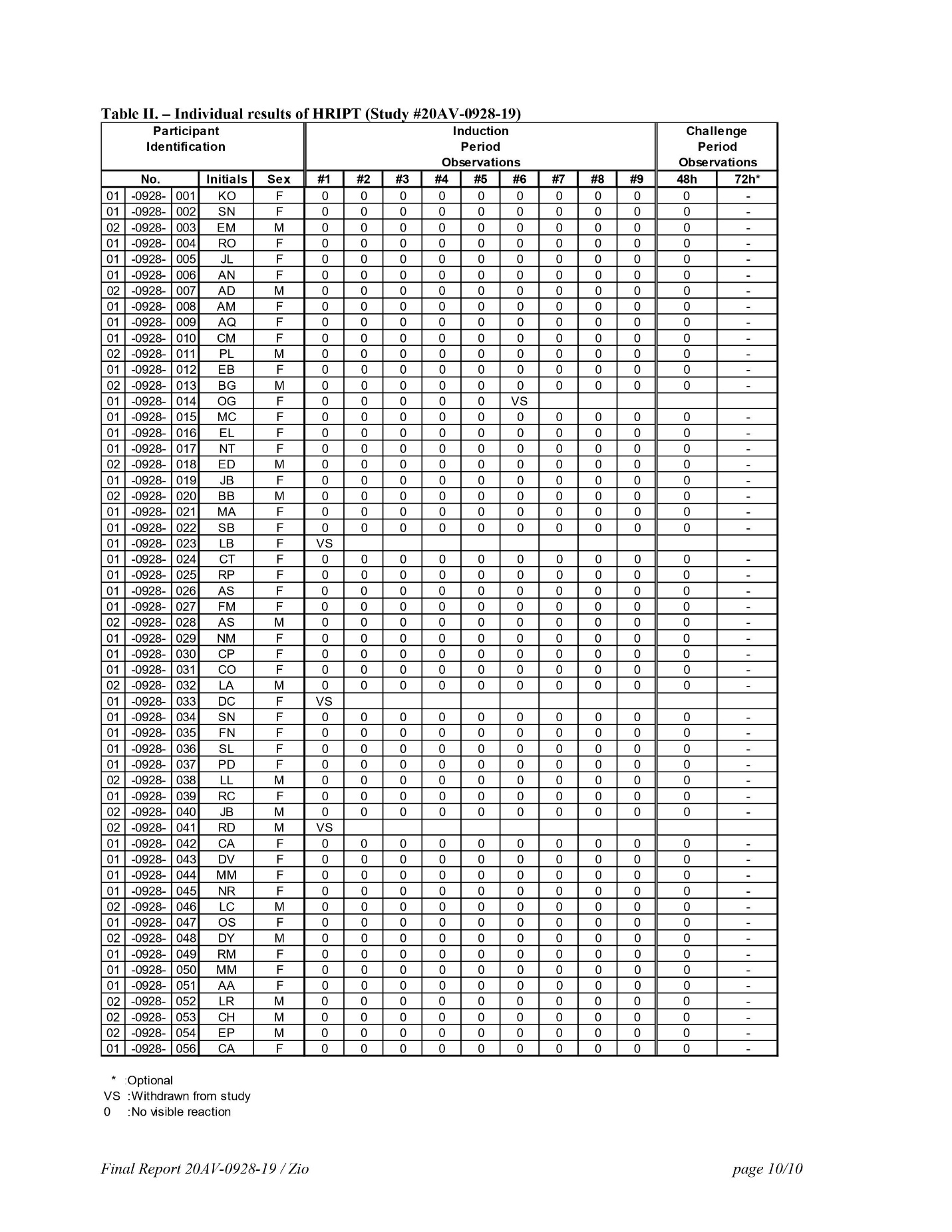

Supplement: Supplementary file 1 — Supplementary Information. [file 41598_2021_3771_MOESM1_ESM.docx]
